# Autoimmune thyroid disease and myasthenia gravis: a study bidirectional Mendelian randomization

**DOI:** 10.3389/fendo.2024.1310083

**Published:** 2024-02-09

**Authors:** Suijian Wang, Kui Wang, Xiaohong Chen, Daiyun Chen, Shaoda Lin

**Affiliations:** ^1^ Department of Endocrinology, The First Affiliated Hospital, School of Medicine, Shantou University, Shantou, China; ^2^ Department of Gastroenterology, The First People's Hospital of Yunnan Province, The Affiliated Hospital of Kunming University of Science and Technology, Kunming, Yunnan, China

**Keywords:** autoimmune thyroid disease, Graves disease, myasthenia gravis, hypothyroidism, Mendelian randomization

## Abstract

**Background:**

Previous studies have suggested a potential association between AITD and MG, but the evidence is limited and controversial, and the exact causal relationship remains uncertain.

**Objective:**

Therefore, we employed a Mendelian randomization (MR) analysis to investigate the causal relationship between AITD and MG.

**Methods:**

To explore the interplay between AITD and MG, We conducted MR studies utilizing GWAS-based summary statistics in the European ancestry. Several techniques were used to ensure the stability of the causal effect, such as random-effect inverse variance weighted, weighted median, MR-Egger regression, and MR-PRESSO. Heterogeneity was evaluated by calculating Cochran’s Q value. Moreover, the presence of horizontal pleiotropy was investigated through MR-Egger regression and MR-PRESSO

**Results:**

The IVW method indicates a causal relationship between both GD(OR 1.31,95%CI 1.08 to 1.60,P=0.005) and autoimmune hypothyroidism (OR: 1.26, 95% CI: 1.08 to 1.47, P =0.002) with MG. However, there is no association found between FT4(OR 0.88,95%CI 0.65 to 1.18,P=0.406), TPOAb(OR: 1.34, 95% CI: 0.86 to 2.07, P =0.186), TSH(OR: 0.97, 95% CI: 0.77 to 1.23, P =0.846), and MG. The reverse MR analysis reveals a causal relationship between MG and GD(OR: 1.50, 95% CI: 1.14 to 1.98, P =3.57e-3), with stable results. On the other hand, there is a significant association with autoimmune hypothyroidism(OR: 1.29, 95% CI: 1.04 to 1.59, P =0.019), but it is considered unstable due to the influence of horizontal pleiotropy (MR PRESSO Distortion Test P < 0.001). MG has a higher prevalence of TPOAb(OR: 1.84, 95% CI: 1.39 to 2.42, P =1.47e-5) positivity and may be linked to elevated TSH levels(Beta:0.08,95% CI:0.01 to 0.14,P =0.011), while there is no correlation between MG and FT4(Beta:-9.03e-3,95% CI:-0.07 to 0.05,P =0.796).

**Conclusion:**

AITD patients are more susceptible to developing MG, and MG patients also have a higher incidence of GD.

## 1 Introduction

Autoimmune thyroid disorders (AITD) emerge from an immune system malfunction, giving rise to an immune onslaught against the thyroid gland (1). AITD stand as the most prevalent autoimmune disorders and hold the position of being the most frequently observed pathological conditions of the thyroid gland (2). This category encompasses two major clinical manifestations: Graves’ disease (GD) and Hashimoto’s thyroiditis (HT), both of which share a common characteristic—lymphocytic infiltration of the thyroid parenchyma (3). The defining clinical traits of GD and HT involve thyrotoxicosis and hypothyroidism, correspondingly (4). The infiltration of the thyroid by autoreactive lymphocytes and the generation of antibodies against three primary thyroid antigens, namely thyroid peroxidase (TPO), thyroglobulin (TG), and thyroid-stimulating hormone receptor (TSHR), are instigated by the activation of T- and B cell pathways (5). AITD’s etiology is presently comprehended as multifactorial, resulting from the intricate interplay between particular susceptibility genes and environmental exposures, with genetic differences and susceptibility playing an important role in the etiology of GD and HT (6).

Myasthenia gravis (MG) exemplifies a classic autoimmune disorder mediated by antibodies, primarily affecting the neuromuscular junction (7). Antibody-mediated processes underlie MG, where antibodies are generated against key components such as the acetylcholine receptor (AchR), the muscle-specific kinase antibody (MuSK), and the agrin receptor low-density lipoprotein receptor-related protein-4 antibody (LRP4) (8).The precise triggering of the autoimmune response in MG remains undisclosed, however, it is evident that deviations within the thymus gland (hyperplasia and neoplasia) have a substantial role, particularly in patients with anti-AChR antibodies (9, 10) and the development of the disorder is plausibly subject to genetic predisposition (11).

MG can coexist with various autoimmune diseases, including autoimmune thyroid disorders, rheumatoid arthritis, systemic lupus erythematosus, type 1 diabetes, and multiple sclerosis (12–14). In MG patients, positive thyroid autoantibodies and antinuclear antibodies are often observed (12). MG and AITD exhibit certain similarities, such as both being organ-specific, antibody-mediated, and contributing to ocular myopathy and exophthalmos (15). Some studies have documented the rising incidence of thyroid disorders in MG, with a higher propensity for MG patients to develop HT and other autoimmune thyroid disorders (16, 17). Patients diagnosed with HT and GD exhibited a heightened subsequent risk of developing MG (18). However, there is a lack of consistency among the reported results, with some studies indicating no clinical association between myasthenia symptomatology and thyroid dysfunction, as well as no significant impact on myasthenic symptoms when the endocrine disorders improve (19). The relationship between AITD and MG is still a topic of ongoing debate, and observational studies are susceptible to the influence of reverse causality and confounding effects. To explore the causal association between AITD and MG, we employed a bidirectional Mendelian randomization (MR) approach in this study. This method utilized genetic variants obtained from genome-wide association studies as instrumental variables (IVs) to mitigate biases commonly found in observational epidemiological studies, such as reverse causation.

## 2 Materials and methods

### 2.1 Study design and the assumption of MR

**Figure 1:**
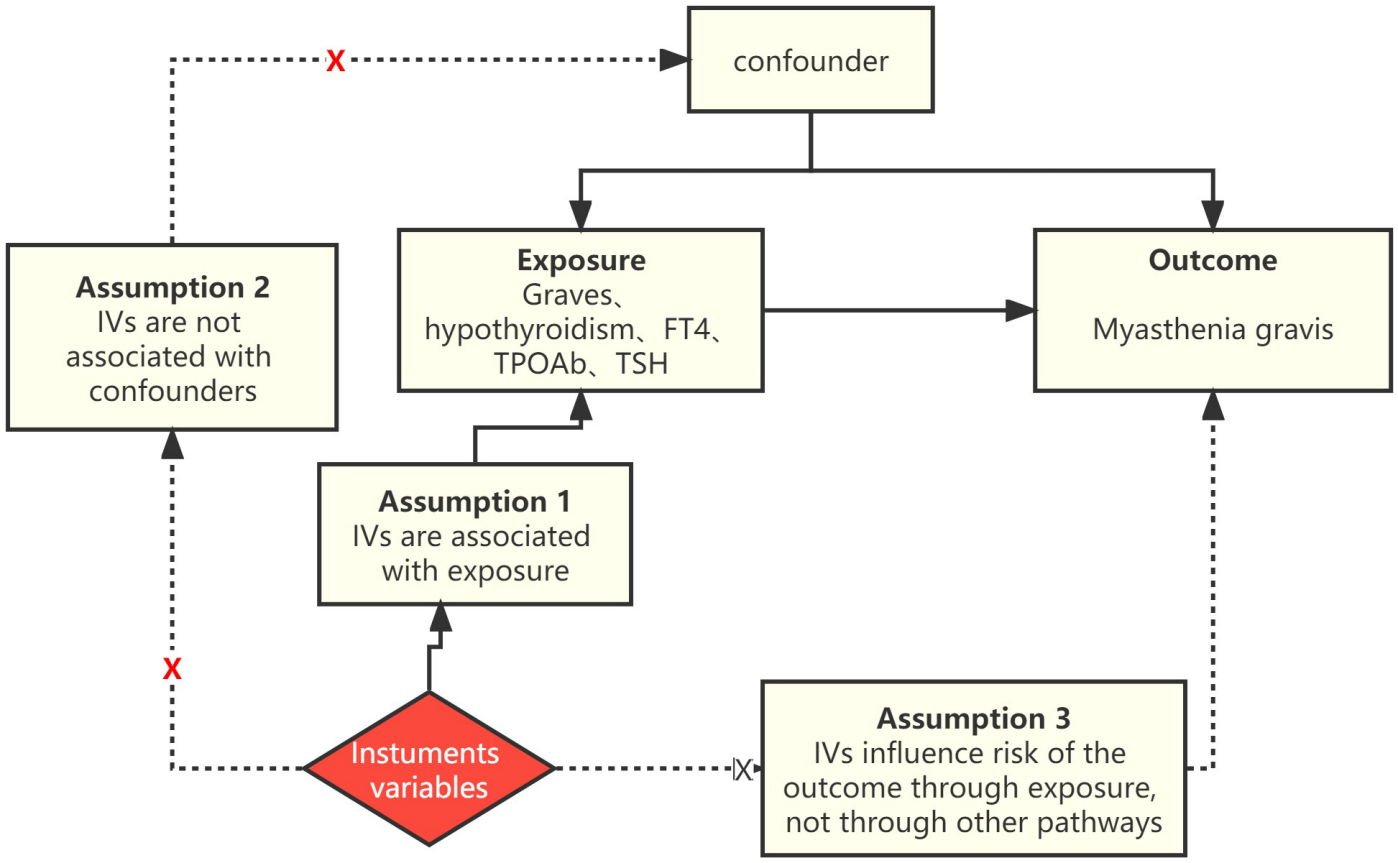
A flow chart outlining the study design and the steps involved in MR analysis.

Employing a two-sample Mendelian randomization (MR) design, we ascertained the overall effects, with the primary objective of assessing the connection between AITD and MG. In a separate two-step MR investigation, we explored whether thyroid function characteristics acted as intermediaries in the impact of AITD on MG. A reverse MR analysis was carried out to assess the reciprocal influence of MG on AITD (**Figure 1**). The MR analysis was conducted under the following assumptions: (i) the single nucleotide polymorphisms (SNPs) used as IVs were obtained from GWAS and displayed associations with the exposures; (ii) the IVs were not associated with confounding factors; (iii) the IVs had an exclusive influence on the risk of outcomes solely through the exposures (20).

### 2.2 Data sources

FinnGen constitutes a substantial collaboration between the public and private sectors, with the objective of gathering and scrutinizing genomic and health information from 500,000 individuals enrolled in FinnGen biobanks (https://www.finngen.fi/en). Within this framework, the FinnGen Biobank of European descent has furnished the Genome-Wide Association Study (GWAS) data associated with ATID, encompassing GD with 4,462 cases and 320,703 controls, as well as autoimmune hypothyroidism with 40,926 cases and 274,069 controls (21). We obtained the summary data for thyroid function GWAS from the ThyroidOmics Consortium, an initiative established to investigate the factors influencing thyroid disorders and thyroid function (22). In a meta-analysis, the analysis of thyroid-stimulating hormone (TSH) included data from 22 distinct cohorts, encompassing a total of 54,288 individuals, while analyses of free thyroxine (FT4) were based on data from 19 cohorts involving 49,269 individuals (22). The GWAS information for thyroid peroxidase antibodies (TPOAb) was extracted from a separate meta-analysis conducted on a general population of 18,297 individuals across 11 different populations. Among these individuals, there were 1,769 cases with TPOAb positivity (23). Samples of individuals with MG were gathered from collaborative sources in both the United States and Italy, constituting a total of 1,873 cases and 36,370 controls (24). The diagnosis of MG relied on established clinical criteria, specifically the presence of characteristic, fatigue-induced muscle weakness, alongside electrophysiological and/or pharmacological anomalies, and further confirmed by the presence of anti-acetylcholine receptor antibodies (25). The complete information is in **Table 1**.

**Table 1:** Details of GWAS included in MR analyses.

Traits	Consortia	Ethnicity	Cases	Control	Sample size	PMID
Graves	FinnGen Biobank	European	4462	320703	325165	36653562
Autoimmune hypothyroidism	FinnGen Biobank	European	40926	274069	314995	36653562
TPOAb	The ThyroidOmics Consortium	European	1769	16528	18297	24586183
FT4	The ThyroidOmics Consortium	European	/	/	49269	30367059
TSH	The ThyroidOmics Consortium	European	/	/	54288	30367059
Myasthenia gravis	HumanOmniExpress arrays	European	1873	36370	38243	35074870

### 2.3 Selection of genetic instrumental variables

To obtain IVs while satisfying the assumption of strong correlation between the exposure and SNPs, we applied a genome-wide significance threshold of P-value (P<5×10-8). Additionally, the datasets were harmonized through the removal of variants in potential linkage disequilibrium (r2 = 0.001, 10,000 kb). Subsequently, we standardized the effect estimates for both exposure and outcome variants and eliminated any potential SNPs with incompatible alleles or palindromic SNPs (26). To assess the strength of genetically determined IVs and avoid any bias towards weak IVs, we used F statistics (beta2/se2) (27) and ensured that F>10 in line with the first MR assumption (28, 29).

### 2.4 Mendelian randomization analyses

The main analysis utilized the inverse-variance weighted (IVW) approach under a random-effects model, which accounts for heterogeneity across SNPs (30). We conducted several sensitivity analyses to ensure the robustness of the primary analysis. The weighted median (WM) method, requiring over 50% of the weight corresponding to valid IVs, was also employed to estimate the causal effects (31). Additionally, we evaluated possible horizontal pleiotropy using MR-Egger intercepts (31, 32). To detect and correct for any potential horizontal pleiotropic outliers, we utilized the MR-PRESSO framework, adjusting the IVW estimate through outlier removal (33). Furthermore, we conducted a leave-one-out analysis to investigate whether the effect estimates were impacted by any singular outlier variant. The analyses were conducted using the R software (version 4.2.3) Two-Sample MR package.

## 3 Result

### 3.1 Forward MR analysis between AITD and MG

After data screening, 24 SNPS were extracted from GD data, 117 SNPS were extracted from the Autoimmune hypothyroidism data, 8 SNPS were extracted from TPOAb data, 41 SNPS were extracted from TSH data, and 19 SNPS were extracted from FT4 data (**Supplementary1 Tables 1**–**5**). The assessment of the effects of these 24 valid IVs on MG consistently revealed a causal association between GD and MG (OR 1.31,95%CI 1.08 to 1.60,P=0.005), and this direction of effect remained consistent when employing both the MR-Egger and Weighted Median methods. Subsequent testing revealed the presence of heterogeneity (Q-pval =1.180e-05), leading to the adoption of a random-effects model to estimate the MR effect size. Neither evidence of horizontal pleiotropy was found through the MR Egger intercept(egger intercept P=0.996), nor was there any significant difference in results after removing two outlier identified by the MR Presso test (MR PRESSO Distortion Test P=0.929).The results remained stable before and after the correction (**Supplementary2 Table 1**). The IVW method, upon analysis, indicated a significant association between autoimmune hypothyroidism and an increased risk of MG (OR: 1.26, 95% CI: 1.08 to 1.47, P =0.002, **Figure 1**). This direction of effect was consistent with the Weighted Median method, and the MR Egger intercept (egger intercept P=0.127) did not reveal any evidence of pleiotropy. After removing six outliers with the MR Presso approach, the results remained unchanged (MR PRESSO Distortion Test P = 0.620). Furthermore, the IVW method found no significant associations between TSH, FT4, and TPOAb with the risk of MG (refer to **Figure 1**). These consistent findings were replicated using alternative methodologies and through replicative analyses (**Supplementary2 Table 1**).

### 3.2 Reverse MR analysis between MG and AITD

**Figure 2:**
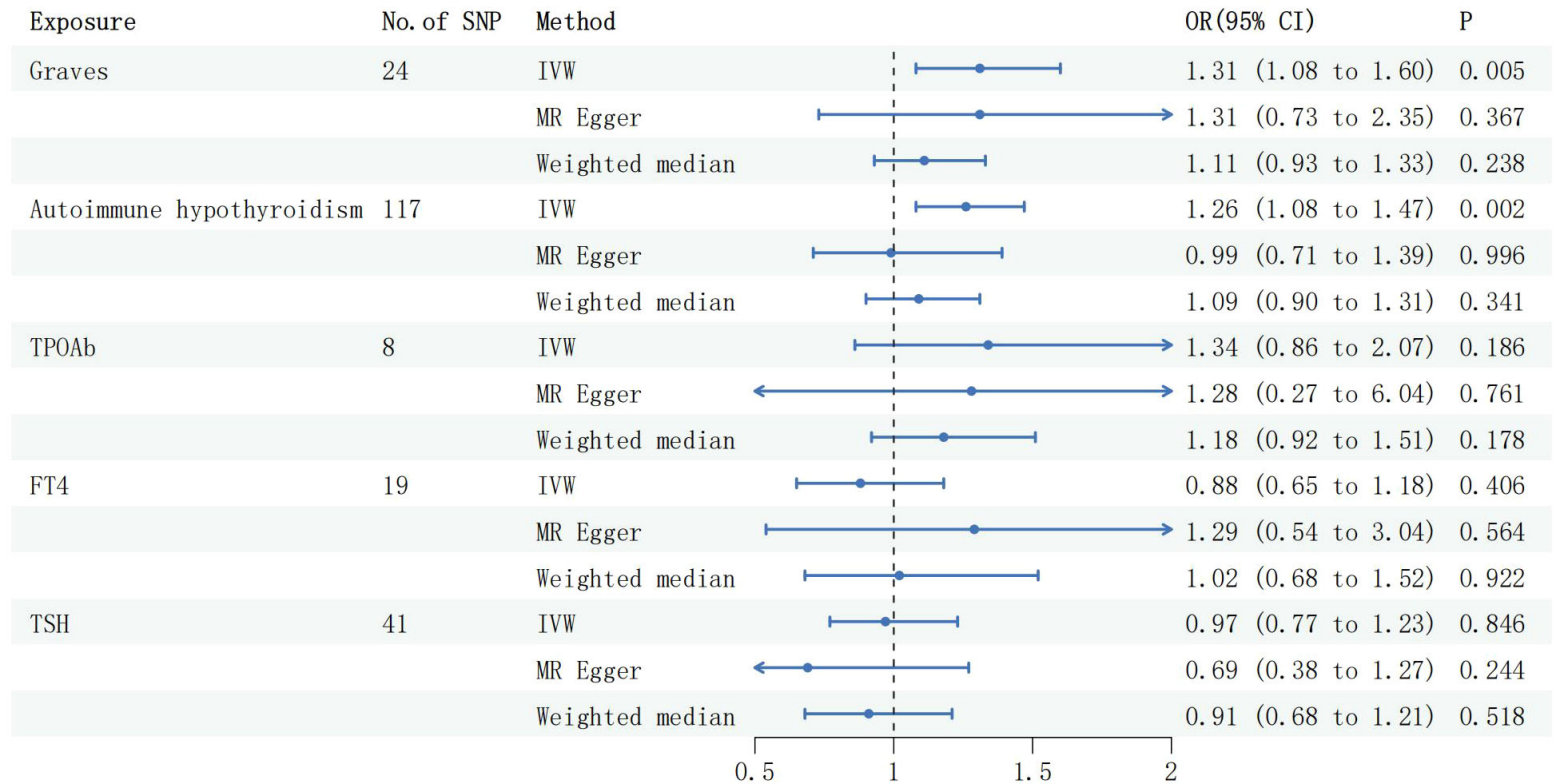
Forest plots of causal effect estimates in forward MR. GD, Graves disease; SNP, single-nucleotide polymorphism; IVW, inverse variance weighted. MG, Myasthenia gravis; TPOAb, thyroid peroxidase antibody; FT4, free thyroxine4; TSH, thyroid stimulating hormone.

During the reverse MR analysis, six SNPs were extracted from the MG dataset and utilized as IVs. The IVW method demonstrated a significant association between MG and an increased risk of GD (OR: 1.50, 95% CI: 1.14 to 1.98, P =3.57e-3, **Figure 2**). This association was corroborated by the Weighted Median method (OR: 1.21, 95% CI: 1.10 to 1.33, P =6.04e-5, **Figure 2**) and the Weighted Mode method (OR: 1.20, 95% CI: 1.10 to 1.31, P =9.30e-3, **Figure 2**). Moreover, the MR Egger intercept (Egger intercept P = 0.310) did not indicate the presence of pleiotropy. After the removal of four outliers with the MR Presso technique, the results remained stable (MR PRESSO Distortion Test P=1). Causal associations were also observed between MG and autoimmune hypothyroidism, supported by the IVW method (OR: 1.29, 95% CI: 1.04 to 1.59, P =0.019, **Figure 2**), the Weighted Median method (OR: 1.12, 95% CI: 1.07 to 1.17, P =7.43e-8, **Figure 2**), and the Weighted Mode method (OR: 1.12, 95% CI: 1.08 to 1.17, P =2.02e-3, **Figure 2**). However, further MR Presso testing revealed the presence of pleiotropy (MR PRESSO Distortion Test P < 0.001), indicating instability in the results.

**Figure 3:**
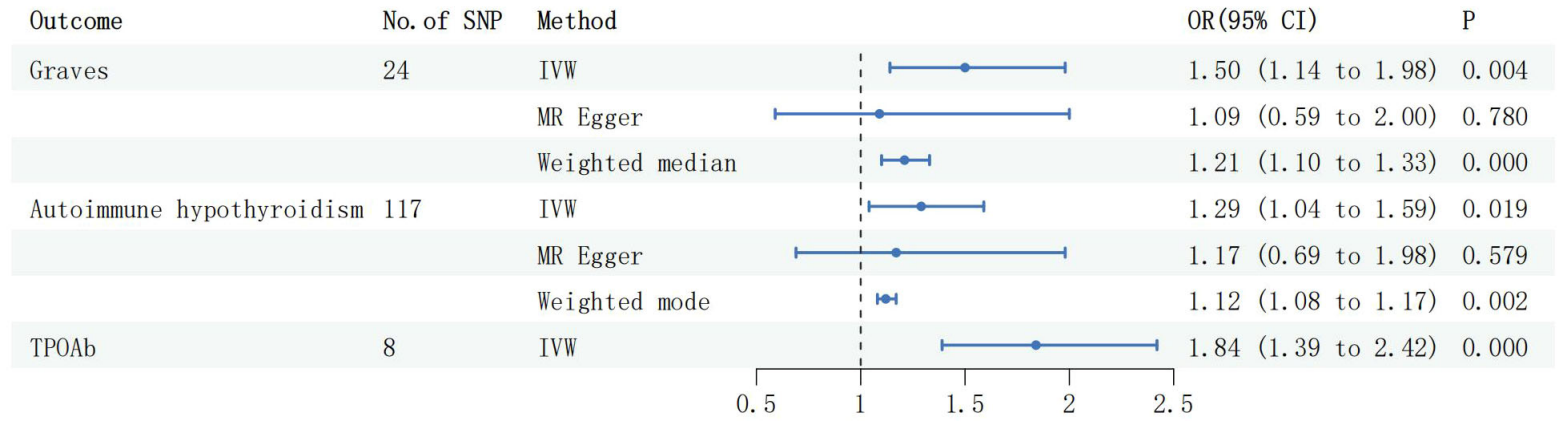
Forest plots of causal effect estimates in reverse MR, GD, Graves disease; SNP, single-nucleotide polymorphism; IVW, inverse variance weighted. MG, Myasthenia gravis; TPOAb, thyroid peroxidase antibody.

**Figure 4:**
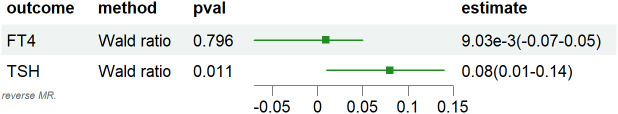
Forest plots of causal effect estimates in reverse MR, IVW, inverse variance weighted. MG, Myasthenia gravis; TPOAb, thyroid peroxidase antibody; FT4, free thyroxine4; TSH, thyroid stimulating hormone.

Following harmonization, a combined total of 2 valid IVs were identified for the association between MG and TPOAb, while 1 valid IV was found for the relationship between MG and FT4/TSH. The IVW method revealed a causal relationship between MG and TPOAb (OR: 1.84, 95% CI: 1.39 to 2.42, P =1.47e-5, **Figure 3**), and the Wald ratio method indicated an association between MG and elevated TSH (Beta:0.08,95% CI:0.01 to 0.14,P =0.011, **Figure 4**), whereas there was no observed correlation with FT4.

### 3.3 F-statistics and visualization of MR

**Figure 5:**
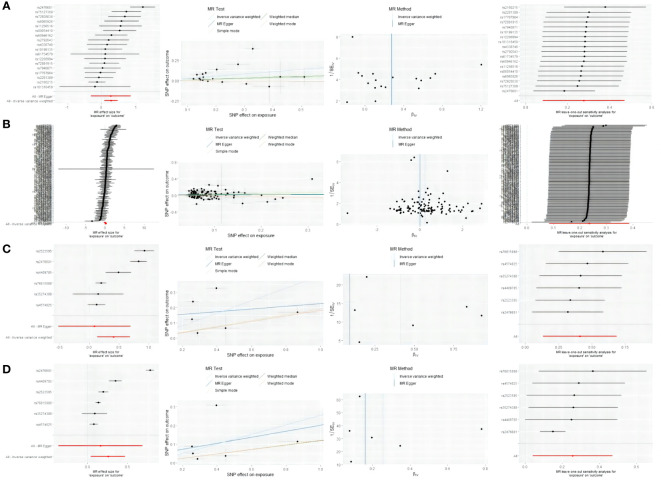
From left to right includes forest plots, scatter plots, funnel plots, and leave-one-out plots showcasing the MR Effect. **(A)** the MR Effect of GD on MG; **(B)** the MR Effect of hypothyroidism on MG; **(C)** the MR Effect of MG on GD; **(D)** the MR Effect of MG on hypothyroidism.

F-statistics were employed to calculate the values for each valid IV, with none of them falling below 10 (**Supplementary1 Tables 9**–**14**). The arrangement of figures from left to right includes forest plots, scatter plots, funnel plots, and leave-one-out plots showcasing the MR Effect (**Figure 5**). The scatter plots exhibit a positive correlation trend between ATID and MG, which is also evident in the reverse MR analysis. The symmetrical funnel plots indicate result stability. The forest plots allow for the observation of the effects of each SNP, while the leave-one-out analysis validates the significance of the results (**Figure 5**).

## 4 Discussion

Previous case reports have described the co-occurrence of GD and MG (34–37). However, the occurrence of thyroid-associated ophthalmopathy(TAO) and MG together is extremely rare. A retrospective study of 1482 MG cases revealed that only 20 cases (1.3%) were identified with TAO (15). The sequence of onset between AITD and MG remains unclear. Studies have reported the TNF-α -863 polymorphism is likely to be associated with MG combined with TAO (36). Both AITD and MG demonstrate a noticeable genetic predisposition (7, 38, 39). Thymoma or thymus hyperplasia is commonly linked to MG (7), and the amelioration of neuromuscular symptoms following thymectomy suggests the involvement of a dysfunctional thymus in the development of MG (40). The presence of thymus hyperplasia in GD was initially described in 1912 and is a prevalent finding (approximately 40% in histology) in patients with thyrotoxicosis (41–43). Multiple lines of evidence indicate that thyroid hormones themselves induce thymus hyperplasia (44–46). In this context, the promiscuous expression of the TSH receptor in thymocytes may be responsible for the autoimmune-mediated expansion of the thymus in GD, facilitated by TSH receptor-stimulating autoantibodies (47). Conversely, it has also been observed that the size of the thymus decreases after thyroidectomy, reflecting the correction of thyrotoxicosis as well as the reduction of the autoimmune response against the TSH receptor (48). Reduction of TPOAb following Thymectomy in Patients with MG (49).

From a pathological perspective, AITD and MG share some common elements, as both pathologies result from a deficient immune response against self-structures. Morphologically, they both exhibit abundant lymphocyte infiltration, mainly distributed in follicles formed by germinal centers (50). Hashimoto thyroiditis and MG show high Ki67 labeling index and strong p63 immunopositivity (51). From an analysis of coexisting antibodies, MG patients often have the presence of thyroid autoantibodies, while MuSK antibody-positive MG patients exhibit coexisting autoimmune diseases, predominantly Hashimoto thyroiditis and rheumatoid arthritis (52). AChR antibody-positive MG patients are associated with one or more coexisting autoimmune diseases, with Graves disease being predominant (52). This immune cross-reactivity may serve as the basis for their comorbidity. From a perspective of genetic susceptibility, both are associated with the HLA gene locus (53). Among them, HLA-DR located on MHC II serves as the main susceptibility gene for AITD and is also related to MG (54). Molecules encoded by this region play a key role in exogenous antigen presentation to CD4+ Th cells, indicating the importance of this pathway in AID initiation and progression (54). The antigen presentation theory of susceptibility to MG proposes that in peripheral lymphoid organs, MG-related self-antigens are processed by B cells, and the MHC II molecules on B cells bind linear antigen peptides on CD4+ T cells’ TCRs (55). The MG-related self-antigen peptide binds to the peptide-binding groove of MHC II molecules through its specific anchoring residues. After B cells are activated by CD4+ T cells and enter the germinal center, they undergo selection for memory B cells or plasma cells that produce antibodies with much higher affinity for self-antigens (55). In AITD, HLA-DR expressed by thyroid follicular cells undergoes the same antigen presentation process (56). The intimacy between HLA II genes and antibody-mediated illnesses is attributed to the joint activation of CD4+ Tcells and stimulated B cells, which is vital for the abundant production of antibodies (when compared to activation by T independent antigens) (57).

It has been proposed that the TSH response to TRH is attenuated in patients with myasthenia gravis, causing an increase in basic TSH reactivity (58). This response might be age-dependent. Additional research suggested that individuals with euthyroid antibody-positivity exhibited a significantly amplified TSH response during TRH stimulation testing, placing them at a heightened risk for thyroid dysfunction (59). Our research findings also suggest that genetic variations contribute to the elevation of TSH in relation to MG. However, further fundamental research is required to elucidate the mechanisms mediating the connection between MG and TSH.

In terms of treatment, FcRn is a major histocompatibility complex (MHC) I-related receptor encoded by the FCGRT gene (60). The blocking of FcRn has been demonstrated to accelerate the clearance of pathogenic IgG, and its inhibition was reported to impair FcRn-mediated antigen presentation and cross-presentation (61). Moreover, blocking FcRn reduces circulating IgG levels and suppresses IgG-IC-mediated immune responses (61). This suggests that targeting FcRn may represent a therapeutic strategy for the treatment of autoimmune diseases. FcRn inhibitors are primarily used for the treatment of MG and have undergone phase 3 clinical trials (62, 63). New evidence from randomized controlled trials (RCTs) also indicates the potential of FcRn inhibitors in the treatment of thyroid-related eye disease (64). After 12 weeks of treatment, the levels of TSHR-Ab and IgG were significantly reduced, and the GO-QoL appearance subscale significantly increased (64). In exploring HT, it was discovered that FcRn expression was lower in HT thyrocytes than in normal thyrocytes (65). Thus, FcRn might contribute to the pathogenesis of HT (65). Although the current evidence is limited, it provides new insights and perspectives for the treatment of MG and AITD, particularly for patients with difficulty distinguishing between myasthenia gravis ocular disease and thyroid-related ocular disease. Further research is needed to clarify whether such treatment should be considered as a priority.

The existence of a correlation between AITD and MG remains a subject of debate. This study utilized MR analysis to provide evidence supporting a causal relationship between AITD and MG based on genetic variation. The findings complement the conclusions drawn from previous observational studies. Our results indicate a higher susceptibility of AITD patients to MG and a greater likelihood of MG patients developing GD. However, the reliability of the results for Autoimmune hypothyroidism is considered questionable due to the influence of horizontal pleiotropy. Furthermore, MG patients exhibit a higher prevalence of TPOAb positivity. Additionally, a positive correlation between MG and TSH is observed, although further validation is required as only one SNP was analyzed.

Our study possessed evident advantages. Firstly, it stood as the inaugural research endeavor to analyze the causal association between AITD and MG using bidirectional two-sample Mendelian randomization.Moreover, the exposure and outcome datasets were sourced from different databases, thereby mitigating the potential interference caused by sample overlap (33). The instrumental variables (IVs) employed in our study were SNPs exhibiting strong associations (P<5e-8) and high intensity (F-statistics > 10). Consequently, the exposure and outcome samples in this study were more comparable, lending greater credibility to our conclusions. Furthermore, our study incorporated a comprehensive sensitivity analysis.

Nonetheless, it is crucial to acknowledge the limitations of our research. Firstly, the available GWAS data for MG and TPOAb is currently restricted, comprising a small number of cases and a limited set of extractable SNPs. To ensure further validation, larger sample sizes of GWAS data are required. Secondly, we did not stratify the causal effects of GD and MG based on gender and age, which may introduce potential heterogeneity due to variations in health status, age, or gender. Moreover, it is worth noting that our study population consisted of Europeans, and therefore, the generalizability of our conclusions to a global population may be limited.

## 5 Conclusion

In summary, our bidirectional two-sample MR analysis explores the relationship between AITD and MG, elucidating the causal associations that retrospective studies fail to address from a perspective of genetic variation. It reveals the bidirectional causal relationship between GD and MG, as well as the causal relationship between hypothyroidism and MG. Furthermore, it indicates a higher prevalence of TPOAb positivity in MG patients, potentially linked to elevated TSH levels. These findings supplement the evidence from previous observational studies.

## Data availability statement

The original contributions presented in the study are included in the article/**Supplementary Material**. Further inquiries can be directed to the corresponding author.

## Author contributions

SW: Conceptualization, Data curation, Formal analysis, Methodology, Resources, Software, Validation, Visualization, Writing – original draft, Writing – review & editing. KW: Data curation, Writing – review & editing, Methodology, Supervision. SL: Conceptualization, Data curation, Formal analysis, Funding acquisition, Methodology, Project administration, Resources, Supervision, Writing – review & editing. XC: Data curation, Methodology, Project administration, Resources, Supervision, Validation, Writing – review & editing. DC: Formal analysis, Investigation, Methodology, Supervision, Validation, Writing – review & editing.
