# Supplementary material for: Autoimmune thyroid disease and myasthenia gravis: a study bidirectional Mendelian randomization
Source: Front Endocrinol (Lausanne). 2024 Feb 9;15:1310083. doi: 10.3389/fendo.2024.1310083 (PMC10884276; doi:10.3389/fendo.2024.1310083)
Supplement: Supplementary file 1 [file DataSheet_1.pdf]

Table 1. Published associations between associated phenotypes of Graves and MG in forward MR

SNP	OA	EA	pval	beta.exposure	se.exposure	eaf.exposure	beta	se	pval
rs10199135	A	G	3.13E-09	-0.188116	0.0317512	0.143784	-0.0208	0.0476	0.663
rs11256516	A	G	5.69E-09	0.132048	0.022667	0.317457	0.0733	0.0418	0.07926
rs12208994	T	G	6.83E-09	0.145765	0.0251537	0.227233	0.0153	0.0452	0.7342
rs17767904	G	A	5.63E-14	0.171135	0.0227682	0.311583	-0.0052	0.0395	0.8961
rs181316459	G	C	1.38E-11	-0.387041	0.057251	0.0477549	0.0724	0.2035	0.7219
rs2160215	T	C	1.64E-42	0.306245	0.0224107	0.296134	-0.0397	0.0384	0.3007
rs2281389	A	G	2.20E-13	-0.2213	0.0301655	0.163543	0.0156	0.0477	0.7445
rs2476601	A	G	1.76E-31	-0.328613	0.0281531	0.855704	0.396	0.0609	7.95E-11
rs2792043	A	C	1.52E-08	0.121001	0.021379	0.501178	-0.0255	0.0367	0.4881
rs4338740	T	C	5.99E-10	0.140786	0.022742	0.31288	0.0235	0.0419	0.5746
rs6065926	A	G	3.29E-11	0.163297	0.024619	0.728487	-0.0949	0.0417	0.02289
rs60946162	C	T	1.37E-12	-0.155383	0.0219261	0.409424	-0.0448	0.0371	0.2273
rs61734579	A	C	1.55E-11	-0.425381	0.063087	0.0389118	-0.0463	0.2164	0.8305
rs72891915	G	A	8.61E-28	0.510963	0.0467639	0.0398985	0.0421	0.1129	0.7089
rs72928038	G	A	2.51E-10	0.202624	0.0320293	0.113825	0.1372	0.0491	0.00518
rs75127309	A	G	7.73E-11	0.276978	0.0425746	0.0582667	0.2013	0.061	0.0009657
rs7940871	C	T	1.73E-11	0.152596	0.022683	0.308396	0.0046	0.0417	0.9124
rs80054410	T	C	5.48E-11	0.15227	0.0232213	0.28246	0.0807	0.0374	0.03088

Table 2. Published associations between associated phenotypes of Hypothyroidism and MG in forward MR

SNP	OA	EA	Pval.x	beta.x	se.x	eaf.x	beta.y	se.y	pval.y
rs10116520	A	G	4.99E-31	0.0922204	0.00796132	0.400977	0.0145	0.0385	0.7068
rs10118880	G	A	5.89E-12	-0.0599654	0.00871311	0.724067	0.0629	0.0395	0.1116
rs10166287	T	G	2.39E-12	-0.0919743	0.0131208	0.102806	-0.0242	0.0508	0.634
rs10259879	A	G	6.65E-12	0.06374	0.00928469	0.231986	-0.0042	0.0418	0.9197
rs10416358	A	G	2.31E-20	0.0792971	0.00857547	0.28634	0.0049	0.0432	0.9096
rs10494077	G	A	2.92E-31	0.109462	0.00941244	0.213715	-0.0891	0.0461	0.05352
rs10514934	T	C	1.20E-13	-0.0823176	0.0110994	0.150875	-0.0429	0.0568	0.4499
rs10748781	C	A	1.20E-21	-0.0784328	0.0082063	0.652488	0.007	0.0367	0.8484
rs10814915	T	C	1.48E-19	-0.0708255	0.00782937	0.543587	-0.0012	0.0369	0.9746
rs10817674	C	A	6.71E-10	0.0491956	0.00796987	0.404202	0.0262	0.0392	0.5043
rs10818050	A	G	9.33E-190	0.24571	0.00836262	0.655429	0.0646	0.0385	0.09312
rs10858026	T	G	3.78E-12	0.06992	0.0100674	0.807437	-0.0156	0.0413	0.705
rs11079035	G	A	4.20E-15	0.0756235	0.00963489	0.202659	-0.0028	0.0479	0.954
rs111352680	G	A	7.13E-11	-0.0550579	0.00844707	0.32381	-0.0881	0.0439	0.04459
rs1131896	G	A	1.37E-25	-0.100287	0.00959097	0.2157	-0.0901	0.0411	0.02842
rs116776245	G	A	3.50E-10	0.0872938	0.0139113	0.0833091	0.0891	0.0806	0.2691
rs116909374	C	T	7.53E-19	-0.212521	0.0239684	0.0306768	0.1546	0.0977	0.1136
rs11694732	G	C	2.43E-44	0.110147	0.0078854	0.42955	-0.0554	0.0373	0.1376
rs11830037	C	A	2.35E-13	0.0996828	0.0136049	0.0888095	-0.0491	0.0677	0.4688
rs11897732	G	A	6.29E-09	0.0463268	0.00797538	0.582497	0.0254	0.037	0.4917
rs11935941	A	C	3.59E-30	-0.141463	0.0123947	0.123206	0.0097	0.045	0.8287

rs11969311	A	C	2.97E-10	-0.0569107	0.00903279	0.252349	0.0614	0.0458	0.1796
rs1203940	T	C	1.13E-25	0.102604	0.00979587	0.792063	-0.0464	0.0439	0.2909
rs12175489	G	A	7.26E-43	-0.151299	0.0110242	0.156735	-0.0755	0.0522	0.1478
rs12206423	C	T	1.61E-08	0.0614412	0.0108764	0.148506	-0.2074	0.058	0.000352
rs1239704	G	A	1.31E-09	-0.0513046	0.00845726	0.691589	-0.0466	0.049	0.3413
rs12407089	G	C	2.32E-28	0.0923609	0.00836234	0.316122	-0.0326	0.0386	0.398
rs12540388	A	T	1.05E-10	-0.0657312	0.0101757	0.1861	-0.0252	0.0433	0.56
rs12697352	G	A	5.24E-10	-0.0499358	0.00803913	0.395907	-0.0043	0.0401	0.9154
rs12756019	G	A	6.53E-13	-0.0576319	0.00801665	0.605562	-0.0255	0.0389	0.5131
rs12897126	T	A	5.37E-17	-0.102215	0.0121998	0.886514	0.0906	0.0495	0.0672
rs12923006	G	A	9.23E-13	0.072125	0.0100994	0.179626	-0.0693	0.0533	0.1935
rs12967678	G	A	5.88E-10	0.0744285	0.0120168	0.117959	0.0764	0.0611	0.2111
rs13137589	A	G	1.17E-14	-0.0631943	0.00818682	0.36779	-0.1278	0.0385	0.0009132
rs1317983	T	C	2.69E-33	0.102264	0.00850572	0.680897	0.0053	0.0398	0.8944
rs1319091	C	A	7.93E-09	-0.0559107	0.00969016	0.795	-0.0427	0.0495	0.3876
rs13447704	T	C	1.28E-08	-0.141098	0.0248049	0.0268399	-0.0722	0.1404	0.6071
rs141686764	A	G	3.33E-08	-0.164372	0.0297604	0.018741	-0.0547	0.1325	0.68
rs1441172	C	T	7.06E-10	-0.0525649	0.00852681	0.31324	0.0255	0.037	0.492
rs144651842	G	A	3.87E-15	0.114059	0.0145133	0.0768931	0.0375	0.7135	0.9581
rs151234	G	C	4.82E-13	0.0851282	0.0117738	0.123296	0.0887	0.0504	0.0786
rs1534424	A	C	1.99E-12	-0.0563343	0.00800768	0.397949	-0.0219	0.0371	0.5553
rs17189980	T	C	8.73E-10	0.159744	0.0260552	0.0219647	-0.1918	0.1123	0.08749

rs17364832	T	G	2.85E-20	0.0788068	0.00854317	0.291179	0.016	0.0407	0.6948
rs17389938	G	C	1.61E-09	0.0665321	0.0110281	0.143917	-0.0216	0.0514	0.6739
rs17447487	C	T	1.44E-08	-0.103313	0.0182239	0.0516195	-0.0452	0.0661	0.4939
rs1808192	A	G	8.01E-09	-0.0459939	0.00797357	0.592061	0.0106	0.0386	0.7844
rs1885013	G	A	3.22E-09	0.0504811	0.00852693	0.693585	-0.1245	0.04	0.001835
rs1990760	C	T	1.07E-15	0.0638177	0.00795928	0.585897	0.0347	0.0371	0.3498
rs200293726	A	T	1.89E-22	-0.0840011	0.00861781	0.305176	0.0061	0.0397	0.8771
rs2046045	T	G	1.81E-58	0.127336	0.00789864	0.414313	0.0463	0.0374	0.2157
rs2049218	C	T	4.19E-37	-0.102774	0.00807538	0.386346	-0.0407	0.0371	0.2724
rs210192	A	G	1.54E-14	0.0728538	0.00948093	0.214912	0.0741	0.0381	0.05182
rs2124594	T	C	2.23E-12	-0.0593882	0.00846101	0.318009	-0.0832	0.0398	0.03653
rs229528	C	T	1.29E-19	0.0721977	0.00796744	0.396414	0.1619	0.0364	8.71E-06
rs244687	A	G	1.08E-16	-0.0741115	0.00893353	0.748077	0.0121	0.0515	0.8148
rs2476601	A	G	8.69E-188	-0.308807	0.0105656	0.854556	0.396	0.0609	7.95E-11
rs2629649	T	G	1.47E-09	0.0507258	0.00838789	0.312967	0.0688	0.0375	0.06623
rs2702968	A	T	1.07E-09	0.0482097	0.00790508	0.428279	0.0711	0.0366	0.0517
rs2739043	A	G	9.56E-12	-0.0551584	0.00809602	0.633324	0.0547	0.0382	0.1523
rs28391281	T	C	1.31E-10	-0.0503426	0.00783427	0.546363	-0.0283	0.0376	0.4514
rs28396553	C	T	2.05E-08	-0.0460976	0.00822089	0.64977	0.0091	0.0368	0.8045
rs2972343	A	G	9.64E-12	-0.0882555	0.0129562	0.105752	-0.0278	0.0664	0.6759
rs2976908	T	G	1.72E-14	-0.0605273	0.00789139	0.483628	-0.0327	0.0375	0.3827
rs30233	G	A	1.07E-11	-0.0540285	0.00794838	0.586573	0.0118	0.037	0.7491

rs3103991	A	G	7.61E-10	0.0558276	0.0090733	0.748361	-0.0315	0.048	0.5118
rs3130186	C	T	1.47E-38	-0.129028	0.00993613	0.198053	-0.0355	0.0425	0.4038
rs322901	C	G	1.62E-08	-0.0445694	0.00789034	0.568082	0.004	0.0366	0.9136
rs35717611	C	T	2.89E-11	0.0538069	0.00808853	0.378383	0.0225	0.0419	0.5919
rs3778752	G	T	9.08E-09	0.0452516	0.0078739	0.439791	0.0188	0.0367	0.6086
rs3946137	A	G	1.85E-08	0.0460636	0.00818831	0.349207	-0.0099	0.0382	0.7952
rs41177	G	A	1.18E-09	-0.048636	0.00799532	0.404602	-0.0071	0.0378	0.8504
rs4409785	T	C	1.60E-17	0.0884395	0.0103381	0.165694	0.2545	0.045	1.54E-08
rs4606850	C	T	5.14E-10	0.0649986	0.0104585	0.165394	-0.0595	0.0456	0.1924
rs4704447	A	G	6.46E-09	-0.0460311	0.00793057	0.56869	-0.0159	0.0367	0.6658
rs4853459	T	C	2.03E-41	-0.123481	0.00915986	0.769568	0.134	0.0417	0.001326
rs4912068	C	G	9.51E-09	-0.0810188	0.0141167	0.0886769	-0.0181	0.0624	0.7719
rs56011703	C	T	2.95E-09	0.0840995	0.0141718	0.0814699	0.0693	0.0505	0.1698
rs56159866	C	T	2.58E-10	-0.0591346	0.0093534	0.232405	-0.0344	0.0437	0.4321
rs568999	G	C	2.59E-10	-0.0533804	0.00844386	0.315823	-0.0967	0.0404	0.01674
rs56983610	C	A	1.34E-18	-0.104236	0.0118418	0.130597	0.075	0.0478	0.1163
rs57652885	C	T	2.16E-09	-0.107083	0.0178911	0.0531312	-0.2298	0.1815	0.2055
rs61201527	A	C	5.00E-08	0.0641356	0.011765	0.124417	-0.0574	0.0576	0.3183
rs61759532	C	T	4.37E-15	0.0778455	0.00992449	0.189239	0.1328	0.0459	0.003801
rs61916675	A	G	9.43E-11	0.0543359	0.00839048	0.316719	0.0042	0.0387	0.9132
rs61938962	C	T	4.09E-22	0.0805646	0.0083324	0.319262	0.034	0.0395	0.3891
rs6471875	C	A	7.93E-10	0.0489914	0.0079709	0.405069	0.0299	0.0372	0.4212

rs66760320	C	T	7.64E-11	-0.0637918	0.00980279	0.202443	0.0335	0.0408	0.4115
rs6724363	G	T	2.38E-28	0.089522	0.00810683	0.624795	0.0955	0.0412	0.02034
rs6831973	T	C	1.36E-12	0.0558804	0.00788404	0.555522	-0.1566	0.0368	2.07E-05
rs7043516	A	C	2.66E-23	-0.106566	0.0107159	0.166287	0.0265	0.0514	0.6068
rs7127620	A	G	3.62E-10	-0.0514586	0.00820742	0.359376	-0.0353	0.0381	0.3544
rs713427	T	C	8.88E-10	0.0574138	0.00936868	0.221866	-0.0047	0.0527	0.9292
rs71430783	G	T	2.43E-20	0.0929704	0.0100599	0.180111	0.1076	0.0431	0.01261
rs7144089	G	C	9.04E-09	-0.0473475	0.00823749	0.445673	-0.0106	0.037	0.7748
rs71641308	C	T	1.46E-08	0.0745947	0.0131656	0.0963323	0.0912	0.0764	0.2323
rs72616654	C	G	7.36E-11	0.051378	0.00788836	0.553338	-0.0162	0.0378	0.668
rs72729322	C	T	1.85E-09	0.0574537	0.0095589	0.209989	0.0811	0.0474	0.0868
rs72751538	G	T	1.11E-09	0.0947907	0.0155558	0.0661338	-0.0837	0.072	0.2452
rs72796365	C	T	3.67E-09	0.141199	0.0239386	0.0260639	-0.0568	0.1377	0.6798
rs735000	C	T	1.25E-10	0.0765716	0.0119025	0.120019	-0.1061	0.0515	0.03916
rs7444908	A	G	6.79E-15	-0.06392	0.00820707	0.356509	0.0202	0.0371	0.5869
rs753760	C	G	3.74E-20	-0.0823464	0.00895538	0.261725	0.0099	0.039	0.8
rs76032549	C	T	6.07E-10	0.0639475	0.0103333	0.170413	0.0256	0.0433	0.5547
rs76169968	G	A	1.14E-10	-0.086105	0.0133555	0.0991748	0.0878	0.0636	0.1673
rs7701443	A	G	8.30E-09	-0.046864	0.00813299	0.380588	0.0245	0.0369	0.5077
rs774121	T	C	2.16E-15	-0.0711606	0.00897138	0.749326	-0.0679	0.0427	0.1117
rs7754251	G	C	2.60E-25	0.0814232	0.00783265	0.447795	0.0087	0.0367	0.8131
rs78783493	C	T	6.00E-09	-0.054713	0.00940616	0.232825	-0.0063	0.0746	0.9324

rs78953577	G	T	1.67E-11	-0.0613847	0.00911757	0.24797	0.0668	0.0467	0.1525
rs7902146	C	T	3.71E-16	-0.0731997	0.0089842	0.750453	0.1013	0.0398	0.01088
rs79051898	C	T	2.58E-08	-0.067706	0.0121605	0.120524	-0.0018	0.0648	0.978
rs79490353	T	C	5.68E-11	0.200239	0.0305624	0.0161635	0.0068	0.1137	0.9521
rs794999	A	G	2.13E-13	-0.0679624	0.00925834	0.771704	-0.0396	0.0438	0.3658
rs8006310	A	G	4.12E-12	-0.0542998	0.00783208	0.504177	0.0486	0.0366	0.1841
rs8193	C	T	1.10E-12	0.058247	0.00818416	0.352685	0.0312	0.0379	0.4114
rs914960	T	A	1.28E-08	-0.0629918	0.0110739	0.150334	-0.1194	0.0544	0.02807
rs9271365	T	G	6.10E-180	0.229767	0.00803286	0.35872	-0.0935	0.0374	0.01246
rs9292	A	G	1.43E-08	-0.124311	0.0219269	0.968429	0.009	0.1464	0.9507
rs9378805	A	C	6.91E-09	0.0454094	0.00783841	0.469179	0.1097	0.0369	0.002921
rs938726	A	C	1.24E-14	-0.0676073	0.00876677	0.73143	0.0018	0.0485	0.9708
rs9497965	C	T	2.29E-15	0.0655856	0.0082763	0.33673	0.0259	0.037	0.4844
rs9981704	C	T	3.13E-08	0.0561317	0.0101429	0.179356	0.1098	0.042	0.008997

Table 3. Published associations between associated phenotypes of TPOAb and MG in forward MR

SNP	EA	OA	beta.x	se.x	pval.x	beta.y	se.y	pval.y
rs10020296	a	g	0.134	0.0293	4.99E-06	0.0037	0.0376	0.9209
rs11602677	a	g	0.1701	0.0347	9.16E-07	0.0617	0.0407	0.1297
rs13021203	a	t	-1.3043	0.282	3.75E-06	0.0816	0.1513	0.5898
rs16999999	t	c	0.3118	0.0619	4.84E-07	0.0352	0.0583	0.5467
rs239935	a	g	0.1294	0.0282	4.47E-06	-0.1051	0.0369	0.004351
rs353648	t	g	0.2442	0.0522	2.92E-06	0.1526	0.0534	0.004297
rs4406425	t	g	0.1458	0.0319	4.73E-06	-0.0281	0.0377	0.4568
rs4766517	c	g	-0.2344	0.0468	5.40E-07	0.0047	0.0371	0.8984
rs6679677	a	c	0.2771	0.0561	7.85E-07	0.3937	0.0613	1.35E-10
rs927221	a	g	-0.2095	0.0447	2.82E-06	0.042	0.0505	0.4055

Table 4. Published associations between associated phenotypes of FT4 and MG in forward MR

SNP	OA	EA	eaf	beta.x	se.x	pval.x	beta.y	Se.y	Pval.y
rs10759944	A	G	0.2157	-0.0769	0.0068	1.37E-29	0.0675	0.0385	0.08001
rs10818937	C	T	0.7921	0.0475	0.007	1.31E-11	0.0256	0.04	0.5216
rs11078333	A	T	0.6799	0.0513	0.0072	9.91E-13	-0.0862	0.0372	0.02047
rs11160652	G	A	0.6332	-0.0579	0.0069	5.91E-17	0.0025	0.0382	0.9471
rs11206244	C	T	0.7143	-0.1148	0.0069	4.78E-63	-0.0121	0.0394	0.7582
rs11873601	G	T	0.94322	-0.1409	0.0166	2.49E-17	0.1237	0.089	0.1647
rs12348139	T	C	0.92903	0.0674	0.0118	1.13E-08	-0.0896	0.0653	0.17
rs225014	T	C	0.5788	0.0535	0.0067	1.83E-15	-0.048	0.0384	0.2108
rs3780190	A	G	0.5559	-0.093	0.0068	3.37E-43	0.0011	0.0365	0.9767
rs4149056	T	C	0.8773	-0.0506	0.0089	1.34E-08	-0.011	0.0514	0.8304
rs4712971	T	C	0.6419	0.0507	0.0074	5.55E-12	-0.0445	0.0427	0.2974
rs55679545	G	A	0.7111	-0.044	0.0076	8.42E-09	-0.0247	0.0412	0.5487
rs56069042	A	G	0.98031	0.1061	0.0186	1.16E-08	-0.0688	0.1038	0.5072
rs67583169	C	G	0.6763	0.0613	0.0095	9.99E-11	0.0338	0.0492	0.493
rs6785807	G	A	0.8823	0.059	0.0093	2.47E-10	-0.0443	0.0505	0.3801
rs6855450	C	T	0.96383	-0.1164	0.0115	2.76E-24	-0.0055	0.0645	0.9323
rs7198944	T	A	0.8136	-0.0516	0.0092	1.70E-08	-0.0373	0.0511	0.4646
rs73405691	A	G	0.91621	0.0556	0.0098	1.61E-08	0.003	0.0573	0.9586
rs78677597	A	C	0.8837	-0.0734	0.0082	4.88E-19	0.0049	0.0417	0.9067
rs951366	T	C	0.647	0.0367	0.0067	4.39E-08	-0.0673	0.0386	0.0809

Table 5. Published associations between associated phenotypes of TSH and MG in forward MR

SNP	OA	EA	eaf.x	beta.x	se.x	pval.x	beta.y	se.y	Pval.y
rs1045476	A	G	0.6314	-0.049	0.0082	2.36E-09	-0.1043	0.0496	0.03539
rs1079418	A	G	0.2821	-0.1009	0.0066	8.23E-53	0.0083	0.039	0.8322
rs10814915	T	C	0.4583	-0.0421	0.0061	5.06E-12	-0.0012	0.0369	0.9746
rs10957494	A	G	0.4089	0.0402	0.0066	1.10E-09	-0.0026	0.0391	0.9476
rs11654194	A	G	0.7244	-0.0467	0.0061	1.82E-14	0.007	0.037	0.8496
rs11755845	C	T	0.2001	-0.0725	0.0069	1.43E-25	0.0385	0.0408	0.3458
rs1203939	A	G	0.7225	0.0493	0.0072	7.48E-12	-0.0435	0.0439	0.3215
rs12217641	C	T	0.1928	-0.0405	0.0066	8.65E-10	-0.0899	0.0395	0.02304
rs12284404	G	A	0.1722	-0.0667	0.0069	2.48E-22	0.045	0.0427	0.2924
rs12410532	C	T	0.1667	-0.1112	0.0085	4.46E-39	0.0836	0.0489	0.08721
rs1265091	C	T	0.1571	0.0571	0.0086	3.20E-11	-0.1083	0.0469	0.02097
rs12950966	C	G	0.3599	0.0523	0.0061	1.29E-17	0.0336	0.0378	0.3746
rs13329353	T	C	0.342	-0.0614	0.0065	5.17E-21	-0.0613	0.0391	0.1173
rs1351283	A	G	0.5064	0.1279	0.0064	9.27E-90	0.0498	0.0381	0.1911
rs1661584	C	T	0.6314	-0.0451	0.007	1.06E-10	-0.0397	0.0436	0.3632
rs17020122	C	T	0.1424	0.1044	0.0114	5.32E-20	-0.176	0.0767	0.02168
rs17477923	T	C	0.1726	-0.0826	0.0069	2.57E-33	0.0291	0.0422	0.4906
rs17767491	A	G	0.2537	-0.0883	0.0065	3.35E-42	-0.0105	0.0395	0.7895
rs1861628	A	G	0.6039	0.0819	0.007	6.13E-32	0.1014	0.0413	0.01407
rs199461	G	A	0.4125	0.0452	0.0074	1.13E-09	0.0556	0.0417	0.1825
rs2268476	A	G	0.3654	-0.0536	0.0072	6.87E-14	0.0103	0.0441	0.8144
rs2272642	A	G	0.136	-0.0442	0.0079	2.10E-08	-0.0456	0.0471	0.333
rs2439301	A	G	0.7596	0.0587	0.0076	8.15E-15	0.0263	0.0445	0.554
rs28502438	T	C	0.228	-0.0338	0.0061	3.70E-08	-0.0138	0.0366	0.7061
rs2928168	G	T	0.4286	-0.1235	0.0095	5.79E-39	0.0216	0.0507	0.6701
rs334719	A	T	0.92995	0.1668	0.0146	3.24E-30	0.0292	0.0837	0.727
rs40393	T	C	0.7285	-0.0475	0.0064	1.00E-13	0.0016	0.0379	0.9662
rs414755	G	A	0.5934	0.0504	0.0061	1.85E-16	-8.00E-04	0.0368	0.9819
rs4578973	C	T	0.2198	-0.0556	0.0074	8.15E-14	-0.0323	0.0434	0.4567
rs4804413	C	T	0.3654	0.0532	0.0062	8.64E-18	0.0023	0.037	0.9503
rs544873	G	A	0.3897	0.05	0.0063	2.53E-15	0.0215	0.0381	0.5729
rs68137036	A	G	0.1992	-0.0847	0.0068	4.09E-35	0.0096	0.0405	0.8132
rs6847675	T	G	0.1896	-0.1147	0.0076	2.56E-51	0.0135	0.0455	0.7664
rs726019	C	T	0.1442	0.061	0.0103	3.39E-09	0.1339	0.0557	0.01622
rs7529705	G	A	0.3439	0.0531	0.0064	1.39E-16	-0.0239	0.0379	0.5284
rs78571365	A	C	0.03938	-0.0868	0.0153	1.49E-08	-0.1325	0.092	0.1499
rs78775620	C	T	0.0087	-0.1719	0.0228	4.78E-14	-0.0075	0.146	0.9592
rs8015085	G	A	0.2097	0.0671	0.0077	2.45E-18	0.1104	0.0427	0.009729
rs9298749	A	C	0.527	0.0393	0.0064	8.80E-10	-0.0046	0.0374	0.9016
rs9497965	C	T	0.3782	0.0444	0.0062	9.81E-13	0.0259	0.037	0.4844
rs9511147	C	G	0.2143	-0.044	0.0065	1.81E-11	0.0043	0.0382	0.9094

Table 6. Published associations between associated phenotypes of MG and Graves in reversee MR

SNP	EA	OA	eaf.x	beta.x	se.x	pval.x	Pval.y	beta.y	se.y
rs2476601	A	G	0.093468086	0.396	0.0609	7.95E-11	1.76E-31	-0.328613	0.0281531
rs2523595	G	A	0.346952383	0.2602	0.039	2.55E-11	2.90E-27	-0.241016	0.022284
rs35274388	A	G	0.037274795	0.4495	0.0812	3.07E-08	0.489959	0.0675976	0.0979145
rs4409785	C	T	0.175914546	0.2545	0.045	1.54E-08	8.66E-06	0.125031	0.0281078
rs4574025	C	T	0.464516382	0.2874	0.0369	7.09E-15	0.106506	-0.0352837	0.0218597
rs76815088	C	T	0.059448788	-0.8661	0.1128	1.58E-14	1.22E-05	-0.171352	0.0391793

Table 7. Published associations between associated phenotypes of MG and hypothyroidism in reversee MR

SNP	EA	OA	eaf.x	beta.x	se.x	pval.x	pval.y	beta.y	se.y
rs2476601	A	G	0.093468086	0.396	0.0609	7.95E-11	8.69E-188	-0.308807	0.0105656
rs2523595	G	A	0.346952383	0.2602	0.039	2.55E-11	1.18E-09	-0.0510994	0.00840002
rs35274388	A	G	0.037274795	0.4495	0.0812	3.07E-08	0.281255	0.0388861	0.0360891
rs4409785	C	T	0.175914546	0.2545	0.045	1.54E-08	1.60E-17	0.0884395	0.010381
rs4574025	C	T	0.464516382	0.2874	0.0369	7.09E-15	0.00495724	-0.0223496	0.00795418
rs76815088	C	T	0.059448788	-0.8661	0.1128	1.58E-14	1.57E-16	-0.114503	0.0138779

Table 8. Published associations between associated phenotypes of MG and TPOAb/FT4/TSH in reversee MR

SNP(TPOAb)	EA	OA	eaf.x	beta.x	se.x	pval.x	beta.y	se.y	pval.y
rs2476601	A	G	0.093468086	0.396	0.0609	7.95E-11	0.2741	0.0541	4.04E-07
rs4409785	C	T	0.175914546	0.2545	0.045	1.54E-08	-0.093	0.0606	0.1247

SNP(FT4)	EA	OA	eaf.x	beta.x	se.x	pval.x	beta.y	se.y	pval.y
rs4409785	C	T	0.175914546	0.2545	0.045	1.54E-08	-0.0023	0.0089	0.7961

SNP(TSH)	EA	OA	eaf.x	beta.x	se.x	pval.x	beta.y	se.y	pval.y
rs4409785	C	T	0.175914546	0.2545	0.045	1.54E-08	0.021	0.0083	0.01119

Table 9. Graves disease F-statistic in forward MR

SNP	R2	F
rs2792043	1.97E-04	63.92210376
rs2476601	4.19E-04	136.2430519
rs11571297	4.22E-04	137.3944337
rs10199135	1.08E-04	35.1017293
rs60946162	1.54E-04	50.22056011
rs9265530	9.67E-05	31.43695847
rs2647004	1.93E-03	627.4445622
rs2281389	1.65E-04	53.81943078
rs72891915	3.67E-04	119.3863813
rs73744913	2.31E-04	75.24150928
rs12208994	1.03E-04	33.58149983
rs61734579	1.40E-04	45.46463963
rs72928038	1.23E-04	40.02065601
rs181316459	1.41E-04	45.70304342
rs2466074	1.22E-04	39.80136112
rs11256516	1.04E-04	33.93695676
rs75127309	1.30E-04	42.32400198
rs7940871	1.39E-04	45.25667491
rs2160215	5.74E-04	186.7344722
rs4338740	1.18E-04	38.32295227
rs17767904	1.74E-04	56.4959885
rs6065926	1.35E-04	43.99597276
rs80054410	1.32E-04	42.99846636
rs1985791	1.94E-04	63.11629189

Table 10. hypothyroidism F-statistic in forward MR

SNP	R2	F
rs10116520	4.26E-04	134.1779291

rs10118880	1.50E-04	47.36443038
rs10166287	1.56E-04	49.13710817
rs10259879	1.50E-04	47.12881769
rs10416358	2.71E-04	85.50588146
rs10494077	4.29E-04	135.2444832
rs10514934	1.75E-04	55.00265288
rs10748781	2.90E-04	91.34775997
rs10814915	2.60E-04	81.83195108
rs10817674	1.21E-04	38.10195773
rs10818050	2.73E-03	863.2929233
rs10858026	1.53E-04	48.23534986
rs11079035	1.96E-04	61.60518527
rs111352680	1.35E-04	42.48389644
rs1131896	3.47E-04	109.335566
rs116776245	1.25E-04	39.37572908
rs116909374	2.50E-04	78.61815658
rs11694732	6.19E-04	195.1170097
rs11830037	1.70E-04	53.68426253
rs11897732	1.07E-04	33.74108764
rs11935941	4.13E-04	130.2599572
rs11969311	1.26E-04	39.69549941
rs1203940	3.48E-04	109.7083796
rs12175489	5.98E-04	188.3541488
rs12206423	1.01E-04	31.91143093
rs1239704	1.17E-04	36.80022635
rs12407089	3.87E-04	121.988225
rs12540388	1.32E-04	41.72648244
rs12697352	1.22E-04	38.583637
rs12756019	1.64E-04	51.68175721
rs12897126	2.23E-04	70.19740963
rs12923006	1.62E-04	51.00088921
rs12967678	1.22E-04	38.36172337
rs13137589	1.89E-04	59.58302851
rs1317983	4.59E-04	144.5508435
rs1319091	1.06E-04	33.29087467
rs13447704	1.03E-04	32.35668323
rs141686764	9.68E-05	30.50530652
rs1441172	1.21E-04	38.00281398
rs144651842	1.96E-04	61.7623791
rs151234	1.66E-04	52.27702144
rs1534424	1.57E-04	49.49138728
rs17189980	1.19E-04	37.58871941
rs17364832	2.70E-04	85.09156902
rs17389938	1.16E-04	36.39639039

rs17447487	1.02E-04	32.13842139
rs1808192	1.06E-04	33.27301021
rs1885013	1.11E-04	35.04850248
rs1990760	2.04E-04	64.28830458
rs200293726	3.02E-04	95.0108841
rs2046045	8.24E-04	259.8932551
rs2049218	5.14E-04	161.9712099
rs210192	1.87E-04	59.04726422
rs2124594	1.56E-04	49.26666342
rs229528	2.61E-04	82.11194968
rs244687	2.18E-04	68.82120127
rs2476601	2.70E-03	854.2464555
rs2629649	1.16E-04	36.57207483
rs2702968	1.18E-04	37.19234499
rs2739043	1.47E-04	46.41703738
rs28391281	1.31E-04	41.29252643
rs28396553	9.98E-05	31.44243693
rs2972343	1.47E-04	46.400805
rs2976908	1.87E-04	58.82918487
rs30233	1.47E-04	46.20466432
rs3103991	1.20E-04	37.8586059
rs3130186	5.35E-04	168.6283706
rs322901	1.01E-04	31.90651585
rs35717611	1.40E-04	44.25211012
rs3778752	1.05E-04	33.0282324
rs3946137	1.00E-04	31.64640944
rs41177	1.17E-04	37.00336685
rs4409785	2.30E-04	72.57907311
rs4606850	1.23E-04	38.624818
rs4704447	1.07E-04	33.68923455
rs4853459	5.77E-04	181.7271629
rs4912068	1.05E-04	32.93839888
rs56011703	1.12E-04	35.21551473
rs56159866	1.27E-04	39.97068165
rs568999	1.27E-04	39.96491025
rs56983610	2.46E-04	77.4813668
rs57652885	1.14E-04	35.82318663
rs61201527	9.43E-05	29.71746114
rs61759532	1.95E-04	61.52446848
rs61916675	1.33E-04	41.93699998
rs61938962	2.97E-04	93.48577493
rs6471875	1.20E-04	37.77654381
rs66760320	1.34E-04	42.34747866
rs6724363	3.87E-04	121.9423788

rs6831973	1.59E-04	50.23641239
rs7043516	3.14E-04	98.89566783
rs7127620	1.25E-04	39.30971173
rs713427	1.19E-04	37.55545398
rs71430783	2.71E-04	85.40814987
rs7144089	1.05E-04	33.0370718
rs71641308	1.02E-04	32.10198397
rs72616654	1.35E-04	42.42073479
rs72729322	1.15E-04	36.12579479
rs72751538	1.18E-04	37.12112837
rs72796365	1.10E-04	34.7906857
rs735000	1.31E-04	41.38627186
rs7444908	1.93E-04	60.65889572
rs753760	2.68E-04	84.55093994
rs76032549	1.22E-04	38.29936072
rs76169968	1.32E-04	41.56867676
rs7701443	1.05E-04	33.2028585
rs774121	2.00E-04	62.91554169
rs7754251	3.43E-04	108.0627796
rs78783493	1.07E-04	33.83401798
rs78953577	1.44E-04	45.32724312
rs7902146	2.11E-04	66.38302246
rs79051898	9.84E-05	30.99907195
rs79490353	1.36E-04	42.92592546
rs794999	1.71E-04	53.88506428
rs8006310	1.53E-04	48.06616497
rs8193	1.61E-04	50.65194704
rs914960	1.03E-04	32.35668976
rs9271365	2.59E-03	818.1485297
rs9292	1.02E-04	32.14118546
rs9378805	1.07E-04	33.56083961
rs938726	1.89E-04	59.47099451
rs9497965	1.99E-04	62.79741767
rs9981704	9.72E-05	30.62593425

Table 11. TPOAb F-statistic in forward MR

SNP	R2	F
rs10020296	1.14E-03	20.91350776
rs11602677	1.31E-03	24.02714689
rs13021203	1.17E-03	21.38992677
rs16999999	1.38E-03	25.37017421

rs239935	1.15E-03	21.05343005
rs353648	1.19E-03	21.88279737
rs4406425	1.14E-03	20.88748772
rs4766517	1.37E-03	25.0828011
rs6679677	1.33E-03	24.39494565
rs927221	1.20E-03	21.96370157

Table 12. TSH F-statistic in forward MR

SNP	R2	F
rs1045476	6.57E-04	35.70659646
rs1079418	4.44E-03	233.7103213
rs10814915	8.77E-04	47.63087083
rs10957494	6.83E-04	37.0978068
rs11654194	1.08E-03	58.60816057
rs11755845	2.03E-03	110.3981591
rs1203939	8.63E-04	46.88272491
rs12217641	6.93E-04	37.65357145
rs12284404	1.72E-03	93.4410019
rs12410532	3.34E-03	171.1412492
rs1265091	9.24E-04	44.08157321
rs12950966	1.35E-03	73.50683231
rs13329353	1.64E-03	89.22653521
rs1351283	7.47E-03	399.3601944
rs1661584	7.64E-04	41.5088789
rs17020122	1.64E-03	83.86374827
rs17477923	2.63E-03	143.2999085
rs17767491	3.39E-03	184.5349765
rs1861628	2.62E-03	136.8847398
rs199461	6.87E-04	37.30761018
rs2268476	1.02E-03	55.41771139
rs2272642	5.76E-04	31.30216354
rs2439301	1.10E-03	59.65310005
rs28502438	5.65E-04	30.70136823
rs2928168	3.17E-03	168.9936315
rs334719	2.55E-03	130.517683
rs40393	1.01E-03	55.08219918
rs414755	1.26E-03	68.26300508
rs4578973	1.08E-03	56.45071606
rs4804413	1.42E-03	73.6246364
rs544873	1.16E-03	62.98583771
rs68137036	2.85E-03	155.1432894
rs6847675	4.36E-03	227.7628887
rs726019	6.46E-04	35.07270163

rs7529705	1.35E-03	68.83543618
rs78571365	6.40E-04	32.18394644
rs78775620	1.17E-03	56.84133072
rs8015085	1.40E-03	75.93597788
rs9298749	6.94E-04	37.70588623
rs9497965	9.44E-04	51.28218975
rs9511147	8.43E-04	45.82079708

Table 13. FT4 F-statistic in forward MR

SNP	R2	F
rs10759944	2.59E-03	127.8842981
rs10818937	9.34E-04	46.0440492
rs11078333	1.03E-03	50.76356425
rs11160652	1.43E-03	70.41113031
rs11206244	5.59E-03	276.8011976
rs11873601	1.46E-03	72.04240131
rs12348139	6.62E-04	32.62407062
rs225014	1.29E-03	63.7588285
rs3780190	3.78E-03	187.0382549
rs4149056	6.56E-04	32.32238437
rs4712971	9.52E-04	46.93910984
rs55679545	6.80E-04	33.51664493
rs56069042	6.60E-04	32.53772988
rs67583169	8.44E-04	41.63476413
rs6785807	8.16E-04	40.24579367
rs6855450	2.08E-03	102.4454442
rs7198944	6.38E-04	31.45618995
rs73405691	6.53E-04	32.18694826
rs78677597	1.62E-03	80.12107823
rs951366	6.09E-04	30.00301459

Table 14. F-statistic in reverse MR

Name	SNP	R2	F
MG-GD	rs2476601	1.10E-03	42.27981454
	rs2523595	1.16E-03	44.51051892
	rs35274388	8.01E-04	30.64253005
	rs4409785	8.36E-04	31.9836359
	rs4574025	1.58E-03	60.6593961
	rs76815088	1.54E-03	58.95154139
MG-AITD	rs2476601	1.10E-03	42.27981454
	rs2523595	1.16E-03	44.51051892
	rs35274388	8.01E-04	30.64253005
	rs4409785	8.36E-04	31.9836359
	rs4574025	1.58E-03	60.6593961
	rs76815088	1.54E-03	58.95154139
MG-TPOAb	rs2476601	1.10E-03	42.27981454
	rs4409785	8.36E-04	31.9836359
MG-FT4	rs4409785	8.36E-04	31.9836359
MG-TSH	rs4409785	8.36E-04	31.9836359
