# Supplementary material for: Autoimmune thyroid disease and myasthenia gravis: a study bidirectional Mendelian randomization
Source: Front Endocrinol (Lausanne). 2024 Feb 9;15:1310083. doi: 10.3389/fendo.2024.1310083 (PMC10884276; doi:10.3389/fendo.2024.1310083)
Supplement: Supplementary file 2 [file DataSheet_2.pdf]

Table1. Forward MR results

(exposure)	(outcome)	Nsnp	Methods	Beta	SE	OR (95%CI)	*P* value	P_{FDR}	Heterogeneity *Q_P* value	Horizontal pleiotrop Egger intercept P	*MRPresso P*
graves	MG	24	MR-Egger	0..27	0.29	1.31（0.73-2.35）	0.367		1.180e-05	0.996	0.929
			Weighted median	0.10	0.09	1.11（0.93-1.33）	0.238				
			Inverse variance weighted	0.27	0.09	1.31（1.08-1.60）	0.005				
			Weighted mode	-0.01	0.11	0.98（0.79-1.22）	0.892				
Autoimmune hypothyroidism	MG	117	MR-Egger	-8.18e-4	0.17	0.99（0.71-1.39）	0.996		2.206e-17	0.127	0.620
			Weighted median	0.08	0.09	1.09（0.90-1.31）	0.341				
			Inverse variance weighted	0.23	0.07	1.26（1.08-1.47）	0.002				
			Weighted mode	-0.16	0.11	0.84（0.67-1.06）	0.152				
TPOAb	MG	8	MR-Egger	0.25	0.78	1.28（0.27-6.04）	0.761		1.461e-08	0.956	-
			Weighted median	0.16	0.12	1.18（0.92-1.51）	0.178				
			Inverse variance weighted	0.29	0.22	1.34（0.86-2.07）	0.186				
			Weighted mode	0.15	0.14	1.16（0.87-1.56）	0.324				
FT4	MG	19	MR-Egger	0.25	0.43	1.29（0.54-3.04）	0.564		0.659	0.366	-
			Weighted median	0.01	0.20	1.02（0.68-1.52）	0.922				
			Inverse variance weighted	-0.12	0.14	0.88（0.65-1.18）	0.406				
			Weighted mode	0.04	0.24	1.04（0.65-1.67）	0.860				
TSH	MG	41	MR-Egger	-0.36	0.30	0.69（0.38-1.27）	0.244		0.029	0.238	-
			Weighted median	-0.09	0.14	0.91（0.68-1.21）	0.518				
			Inverse variance weighted	-0.02	0.11	0.97（0.77-1.23）	0.846				
			Weighted mode	-0.07	0.21	0.92（0.60-1.40）	0.716				

Nsnp, number of snps.

nominal significant estimate is defined as *P* value < 0.05.

Cochran's Q-derived *P* value and MR-Egger intercept-derived *P* value < 0.05 is significant.

Table2. Reverse MR results

(exposure)	(outcome)	Nsnp	Methods	Beta	SE	OR /Beta(95%CI)	*P* value	P_{FDR}	Heterogeneity *Q_P* value	Horizontal pleiotrop	
										Egger intercept P	*MRPresso P*
MG	graves	6	MR-Egger	0.08	0.30	1.09（0.59-2.00）	0.78		5.407e-22	0.310	1
			Weighted median	0.19	0.04	1.21（1.10-1.33）	6.04e-5				
			Inverse variance weighted	0.41	0.14	1.50（1.14-1.98）	3.57e-3				
			Weighted mode	0.18	0.04	1.20（1.10-1.31）	9.30e-3				
MG	Autoimmune hypothyroidism	6	MR-Egger	0.16	0.26	1.17（0.69-1.98）	0.579		1.025e-105	0.716	0.001
			Weighted median	0.11	0.02	1.12（1.07-1.17）	7.43e-8				
			Inverse variance weighted	0.25	0.10	1.29（1.04-1.59）	0.019				
			Weighted mode	0.12	0.02	1.12（1.08-1.17）	2.02e-3				
MG	TPOAb	2	Inverse variance weighted	0.61	0.14	1.84（1.39-2.42）	1.47e-5		-	-	-
MG	FT4	1	Wald ratio	-9.03e-3	0.03	-9.03e-3（-0.07-0.05）	0.796		-	-	-
MG	TSH	1	Wald ratio	0.08	0.03	0.08（0.01-0.14）	0.011		-	-	-
